# Lipid-Bilayer-Spanning DNA Nanopores with a Bifunctional Porphyrin Anchor**

**DOI:** 10.1002/anie.201305765

**Published:** 2013-09-06

**Authors:** Jonathan R Burns, Kerstin Göpfrich, James W Wood, Vivek V Thacker, Eugen Stulz, Ulrich F Keyser, Stefan Howorka

**Affiliations:** Department of Chemistry, University College London20 Gordon Street, London WC1H OAJ (UK); Cavendish Laboratory, University of CambridgeCambridge CB3 0HE (UK); School of Chemistry, University of SouthamptonSouthampton SO17 1BJ (UK)

**Keywords:** DNA structures, membranes, nanopores, porphyrins, single-molecule studies

Chemistry is in a powerful position to enhance the capabilities of DNA nanotechnology.[1,2] DNA origami offers rationally designed nanoscale structural frameworks,[2,3] yet chemistry can advance them by adding tailored functionality through the selective modification of nucleic acids with chemical tags. This synergistic approach has helped create new nanoscale devices, ranging from affinity nanoarrays capable of binding proteins[4] and quantum dots,[5] to nanoplatforms for single-molecule photochemistry,[6] and to molecular display agents for biosensing.[7,8] Herein, we present a unique chemical strategy for enlarging and enriching the emerging class of membrane-spanning nanopores composed of folded DNA.[9,10] We show that solely two porphyrin-based hydrophobic tags achieve the otherwise energetically unfavorable anchoring of the highly negatively charged DNA nanostructure into the hydrophobic core of lipid bilayers. This very small number of porphyrin tags considerably simplifies the currently available chemical strategies for bilayer anchoring of nanopores. The aromatic porphyrin tags are also fluorescent, and hence facilitate the microscopic visualization of DNA-based membrane channels. Our generic route for dual-functional chemical tags can likely be applied to many other DNA designs, and will help broaden experimental access to versatile DNA origami pores.

Membrane-spanning nanopores composed of folded and structurally defined DNA are the most recent and striking example of a long series of artificial or synthetic membrane channels,[11] including those made of porphyrins.[12] In general, biomimetic and engineered nanopores are of scientific and biotechnological interest, because they are able to replicate the transport of water-soluble molecules across bilayers[13] for applications in research or biosensing.[14] Inspired by nanofunnels[15] and porous nanoplates,[16] DNA-origami pores have been designed to insert into lipid bilayers.[9,10] In these studies, hydrophobic chemical tags were deliberately positioned to anchor the strongly hydrophilic DNA structures into bilayers. The tags previously described were either cholesterol-based lipid anchors covalently attached to DNA strands,[9] or ethyl-modified phosphorothioate groups that replace the negatively charged backbone phosphate to form a hydrophobic belt to mimic natural protein pores.[10] The former tag was placed at up to 26 strategic positions of the pore, whereas the latter group was introduced 72 times into a DNA origami structure. With the intent to simplify nanopore design, and move towards minimal chemical intervention, the present study explores whether other chemical tags of greater hydrophobicity can achieve membrane anchoring using a very small number of tag copies.

We surmised that a porphyrin derivative would satisfy the criterion of strong hydrophobicity because of its large aromatic core. Porphyrin has a van der Waals surface area approximately 12 times higher than ethane, and the area can be further increased with additional aromatic substituents. In addition, most porphyrins are chromophores with a fluorescence emission at 656 nm and can thereby act as powerful visualization tags. Moreover, inserting porphyrins into lipid bilayers leads to a characteristic shift in their fluorescence spectrum, which offers an additional experimental handle to confirm membrane anchoring.[17,18]

For the creation of membrane-spanning DNA nanopores, we selected the tetraphenylporphyrin (TPP) tag (Figure 1 a)[19,20] which matches the requirements in terms of hydrophobicity and fluorescence emission and can be easily coupled to DNA.[21] Acetylene-TPP was attached to deoxyuridine through a Sonogashira coupling to achieve a rigid linkage. The site-specific insertion of the modified nucleoside into oligo-deoxyribonucleotides was accomplished using standard phosphoramidite chemistry as previously described (Figure 1 a; see also the Supporting Information, Figures S1–S4).[19,21]

**Figure 1:**
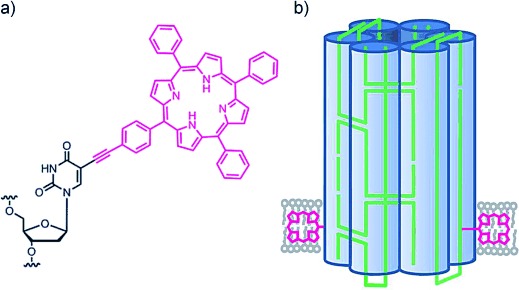
DNA-nanopore-carrying porphyrin-based lipid anchors. a) Deoxyuridine bonded to tetraphenylporphyrin (TPP) through an acetylene linkage at the 5 position of the nucleobase. b) A DNA nanopore composed of six interconnected duplexes, represented as cylinders. The six-component DNA oligonucleotides are shown in green. The magenta porphyrin tags anchor the DNA nanopore into the lipid bilayer. For reasons of visual clarity, only the porphyrin core without the phenyl groups is shown. The TPP tags are not drawn to scale.

In our nanopore design (Figure 1 b), a total of six DNA oligonucleotides were folded into six DNA duplexes, which are interconnected by crossovers to add structural stability. The threading of the oligonucleotides through the duplexes is indicated by the green lines in Figure 1 b (see also Figure S5). The resulting six-helix bundle has a width of 5.5 nm, a height of 14 nm, and an inner channel diameter of approximately 2 nm. The overall dimensions are close to those of our previously published nanopore,[10] but the current design is considerably simpler, as six rather than 14 strands are used. Crucially, two porphyrin tags are positioned at the end of the nanobarrel to achieve its insertion into the bilayer in a directional manner (Figure 1 b). To facilitate the comparison to our previous nanopore work, we used a design with two DNA strands that contain phosphorothiate groups in place of the phosphates in a number of positions in the backbone. The thioate groups behave like the native negatively charged phosphate groups at our experimental conditions of pH 8.0 (Table S1).[10]

The DNA nanopore was assembled by heating and cooling an equimolar mixture of four regular and two TPP-modified DNA strands (for sequences see Table S1). The assembly mixture was characterized to confirm the correct and successful formation of the DNA nanopore. Native gel electrophoresis yielded a band, which migrated to the same height as a control nanopore without the porphyrin anchor (Figure 2 a, lanes 2 and 3, respectively; main band co-migrating at the 550 bp marker). The tailing of the band for the porphyrin DNA pore does not indicate unfolding, but is rather caused by the very hydrophobic tag.[9,19,20] The concerted assembly into the nanobarrels was also confirmed by a single defined transition in the UV melting profiles, (Figure 2 b; *T_m_*=53.4±1.0 °C; *n*=3); the opposite and unexpected independent hybridization of the multicomponent DNA duplexes of different melting temperatures would have led to a very broad transition. Furthermore, dynamic light scattering (DLS) established the monomeric nature of the nanobarrels, as only a single peak with a hydrodynamic radius of 5.5±0.1 nm was observed (Figure 2 c). The radius is larger than the calculated value of 4.9 nm,[22,23] but in line with the accuracy of DLS measurements for related DNA nanostructures.[23,24] The detailed dimensions of the nanobarrel were established with atomic force microscopy (AFM) analysis (Figure 2 d). The apparent height of (2.20±0.25) nm was expected for tip-compressed hollow DNA nanostructures.[8,25] Similarly, the AFM-derived length and width of (20.4±4.5) nm and (9.7±2) nm (full width at half maximum), respectively, were in good agreement with the theoretical dimensions (14 nm and 5.5 nm) after correcting for tip-deconvolution.[10,26]

**Figure 2:**
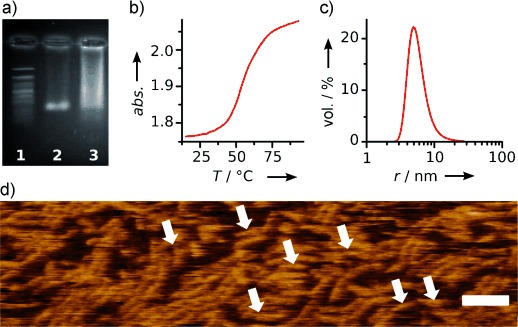
Characterization of porphyrin-modified nanobarrels assembled from DNA oligonucleotides. a) Agarose gel electrophoresis. Lane 1, 100 bp marker; lane 2, DNA nanopore; lane 3, TPP-DNA nanopore. b) UV melting profile of a TPP nanopore. c) Dynamic light scattering trace of TPP nanopore. d) AFM micrograph of individual nanopores (arrows), and of elongated nanopore assemblies, which are stabilized by inter-barrel base stacking; scale bar=50 nm. *abs.*=absorbance, *T*=temperature.

Having completed their structural characterization, we investigated whether the porphyrin-tagged DNA nanopore can be stably anchored into lipid bilayers. In this examination we took advantage of the fluorescence properties of the porphyrin tag. Once incubated with giant unilamellar vesicles, the tagged nanopores could be visualized using fluorescence microscopy (Figure 3 a, bottom). A bright-field image documented the shape of the vesicle (Figure 3 a, top). The bright fluorescence spots in the ring-shaped bilayer may represent individual DNA nanopores, given that the imaging conditions of our custom-built set-up are powerful enough to detect single fluorophores.[27] However, as the DNA pores rapidly diffuse out of the focal plane (Supporting Information, Movie SM1) and exhibit surprising photostability, it is hard to detect individual pores by single-fluorophore photobleaching. The anchoring of the nanopore into the bilayer was confirmed by independent spectroscopic analysis based on the known fluorescence shift of porphyrin. As illustrated in Figure 3 b, the fluorescence emission maxima at 605 nm and 653 nm shifted upon bilayer insertion, by 2 nm and 1 nm, respectively. These changes are in line with previous studies on the membrane binding of individual porphyrin-carrying DNA duplexes[17] and were enhanced by the use of zinc-porphyin tags (Figure S6). Membrane-anchoring also led to an increase of the thermal stability of the DNA nanopore by almost 6 °C to a *T_m_* of 59.2±1.2 °C, as determined by UV-based melting profiles (data not shown). The fluorescence measurements do not, however, rule out that a proportion of the DNA nanobarrels anchor with only one TPP group and lie parallel to the membrane plane.

**Figure 3:**
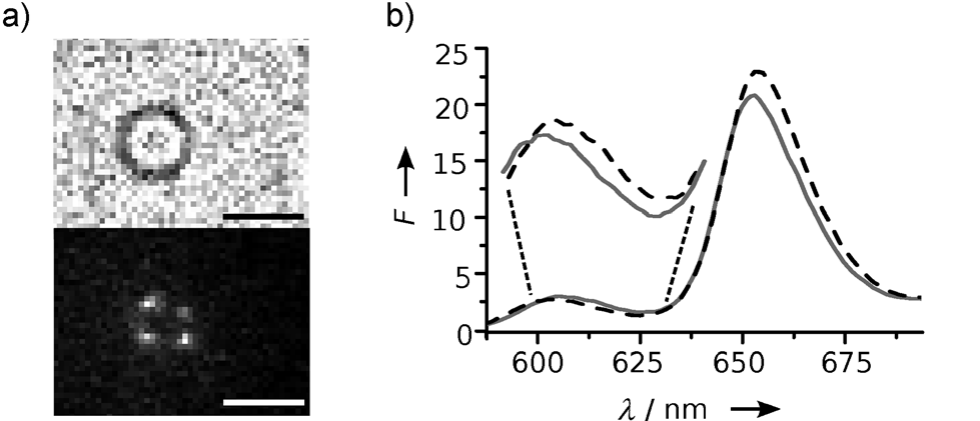
Porphyrin tags anchor DNA nanopores into lipid bilayers. a) Microscopic bright-field (top) and fluorescence (bottom; *λ*_exc_=532 nm) images of a DPhPC vesicle containing DNA nanopores; scale bars=5 μm. b) The fluorescence emission spectrum for the TPP nanopore (—) is shifted upon insertion into vesicle bilayers (- - - -; *λ*_exc_=424 nm). *F*=fluorescence intensity.

To provide evidence that the porphyrin nanopore is capable of spanning the membrane, we carried out current recordings to measure the ion flow across the lipid bilayers. Lipid vesicles were first incubated with DNA nanopores, then suctioned onto a nanocapillary, and, after application of a voltage pulse, analyzed by recording the ionic current caused by the transmembrane potential. A representative current trace for a single pore at standard electrolyte conditions and potentials (Figure 4 a) demonstrates that the inserted pores punctured the bilayer with nanoscale holes of steady ionic current. Results from multiple nanopore recordings yielded a distribution of conductances with a maximum of 250 pS (Figure 4 b and 4 d). This value is slightly lower than the average obtained from nanopores with a similar six-helix core structure,[10] which could be due to the divergent designs, as the present nanopore inserts at its terminus, whereas the previously reported one crosses the bilayer at the middle section. In combination with the lateral membrane pressure of the capillary-held vesicles, slightly different inner-channel cross-sections could result. Alternatively, the pore might insert into the membrane in an angled rather than the assumed perpendicular orientation, thus leading to partial obstruction of the ion conduction path. These factors might also contribute to the observed broad distribution of conductances (Figure 4 b), which could additionally be due to inferring of single-pore conductances from multistep closures (Figure 4 d) with a potentially different minimal residual current, or to some structural variability of the assembled DNA network, as the RMS current noise changed between channels (not shown). However, under additional analysis, the porphyrin nanopores exhibited almost ohmic behavior in current–voltage curves (Figure 4 c), which implies overall structural integrity within the DNA nanoarchitecture and stable insertion into the lipid bilayer.

**Figure 4:**
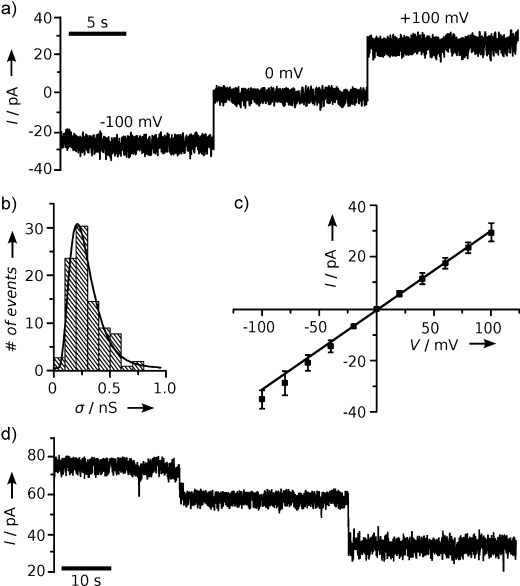
Porphyrin-DNA nanopores span lipid bilayers. a) Current trace of membrane-inserted TPP nanopores in 1 m KCl, 50 mm Tris, pH 8.0, acquired at a filtering and sampling frequency of 2 kHz and 10 kHz, respectively. A prolonged trace is shown in Figure S7. The RMS noise increases from 1.30 pA to 1.43 pA from 0 mV to 100 mV. b) Histogram of single-pore conductances, as determined from single-channel recordings or from current steps of multichannel traces (panel d). c) Voltage–current curve, obtained from 8 independent recordings. d) Current trace of multiple pores, with the stepwise reduction interpreted as separate pore closures.

In summary, our overarching research objective to synergistically combine chemistry with DNA nanotechnology has led to the creation of membrane-spanning DNA nanopores that carry two bifunctional tags. Exemplified by a relatively simple nanostructure, our strategy of minimal chemical intervention drastically increases flexibility in nanopore design and will enable more complex architectures that can perform higher-level functions for applications in biomimetic research and biosensing.

## Experimental Section

Design of the nanopore structure: The nanobarrel was designed using the caDNAno software.[28] Several suggested scaffold and staple strands were terminally linked to form a more stable structure composed of only six DNA strands (Figure S5). Using a molecular model, which was generated with Macromodel in combination with caDNAno, the positions for attaching porphyrins were selected to be on two opposite duplexes.

Synthesis and purification of porphyrin-DNA, and nanopore assembly: Tetraphenylporphyrin-tagged deoxyuridine was synthesized and incorporated into DNA oligonucleotides as described (Figures S1 and S2).[19] The purity of the strands was confirmed by PAGE, whereas the yield was determined by UV/Vis spectroscopy (Figures S3 and S4). Nanopores were assembled by heating an equimolar mixture of the six strands (1 μm each), dissolved in buffer **A** (KCl (1 m), tris(hydroxymethylaminomethane) (Tris; 50 mm, pH 8.0); total volume=1000 μL) at 95 °C for 5 min, followed by cooling to 16 °C at a rate of 0.25 °C min^−1^ in a Varian Cary 300 Bio UV/Vis spectrophotometer, equipped with a Peltier cooling element.

Nanobarrel characterization was accomplished with native gel electrophoresis, UV/Vis spectroscopy, dynamic light scattering, and atomic force microscopy. The assembled DNA barrels were analyzed using agarose (0.8 %) gel electrophoresis in a standard TBE buffer, supplemented with MgCl_2_ (11 mm) and running conditions of 80 V, 80 min, 8 °C, followed by ethidium bromide staining. The melting point analysis was performed on samples (0.2 μm) dissolved in buffer **A**, using a heating rate of 0.5 °C min^−1^. DLS experiments were conducted on a Zetasizer Nano S from Malvern,[29] using DNA samples (0.25 μm) in buffer **A**. AFM analysis was carried out by first adsorbing DNA barrels onto mica, following a modified version of a published procedure.[30] Freshly cleaned mica was incubated with a solution of MgCl_2_ (3 mm) for 5 min. The surface was then incubated with a 20 nm solution of the DNA-barrel solution. AFM topographical images were acquired in situ at RT with a Multimode atomic force microscope as described.[10]

Lipid vesicle formation: Giant unilamellar vesicles (GUVs) for imaging and current recordings were prepared using an electroformation unit (Vesicle Prep Pro, Nanion Technologies, Germany) as described.[31] The GUVs (1–30 μm) were stored at 4 °C for up to a week.

Ionic current recordings: The recordings were performed using a nanobilayer setup as previously described.[32] On this setup, the bilayers were formed reproducibly by bursting GUVs on the tip of a nanocapillary (200 nm diameter). GUVs were incubated with DNA nanopores (30 nm) for 1 h at RT in buffer **A**. Bilayers that held DNA nanopores were identified by their lowered seal resistances. Nanopore incorporation was triggered by applying a voltage pulse. Ionic current data were acquired using an Axopatch 200B amplifier and analyzed as described.[32,33]

Fluorescence microscopic imaging: DNA nanopores (3 nm) were incubated with GUVs (0.5 μL) in buffer **A** for 1 h at RT. The suspension (20 μL) was then imaged using a previously described microscope setup[34] with a laser (λ=532 nm, 2 mW) and an EMCCD camera (acquisition time=5 ms). Data acquisition and image processing were performed as described.[34]
