# Supplementary material for: Lipid-Bilayer-Spanning DNA Nanopores with a Bifunctional Porphyrin Anchor
Source: Angew Chem Int Ed Engl. 2013 Sep 6;52(46):12069–72. doi: 10.1002/anie.201305765 (PMC4016739; doi:10.1002/anie.201305765)
Supplement: Supplementary file 2 [file anie0052-12069-sd2.pdf]

Angewandte Chemie
Eine Zeitschrift der Gesellschaft Deutscher Chemiker

Supporting Information

© Wiley-VCH 2013

69451 Weinheim, Germany

Lipid-Bilayer-Spanning DNA Nanopores with a Bifunctional Porphyrin Anchor**

*Jonathan R. Burns, Kerstin Göpfrich, James W. Wood, Vivek V. Thacker, Eugen Stulz, Ulrich F. Keyser, and Stefan Howorka**

anie_201305765_sm_miscellaneous_information.pdf
anie_201305765_sm_dna_nanopores_movie.avi

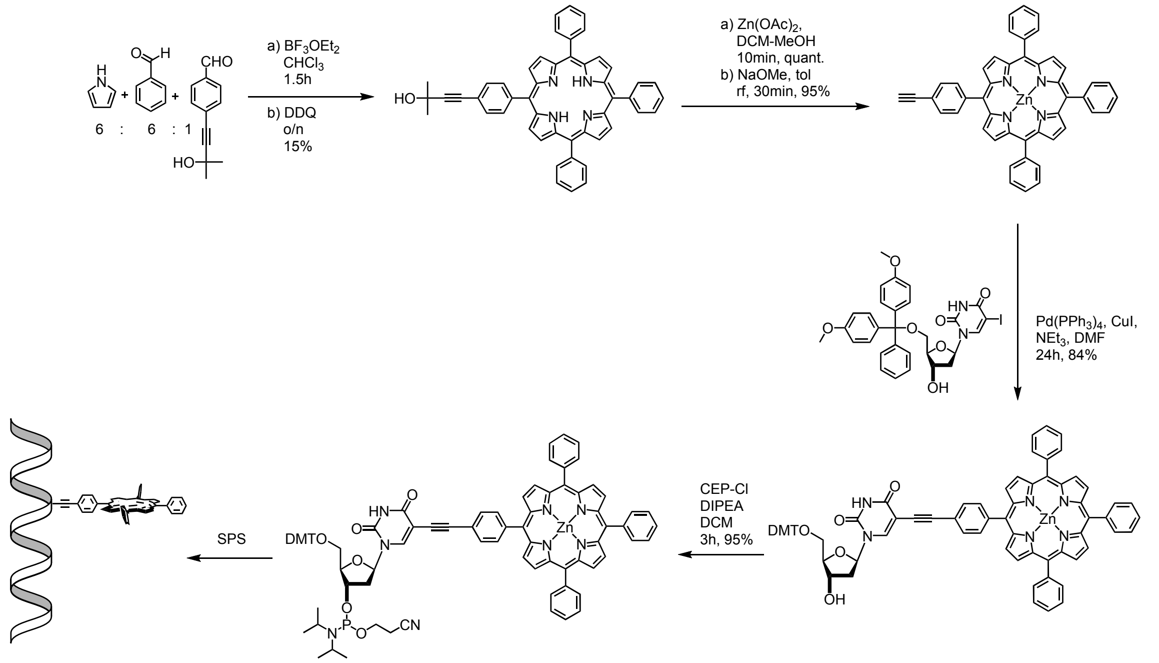

Figure S1. Synthesis of porphyrin-dU building block.[1] The protected acetylene benzaldehyde was synthesised according to literature procedures.[2-3] The use of an excess of pyrrole and benzaldehyde suppresses formation of higher substituted TPP in the porphyrin synthesis. The phosphoramidite porphyrin-dU is highly sensitive to oxidation; the product was precipitated from the reaction mixture with hexane, washed with hexane and directly used for DNA synthesis under inert atmosphere. Solid phase synthesis (SPS) of the DNA followed standard phosphoramidite chemistry for the unmodified parts of the ODN using an Applied Biosystems Expedite DNA synthesizer (1000 Å CPG) with reagents obtained from Tides Service, Haar, Germany. For insertion of the dU-TPP building block, a freshly made solution of the building block in DCM-MeCN 1:1 (50 mM) was used with an extended coupling time (10 min), otherwise using standard SPS protocols. The modified DNA was cleaved from the CPG and deprotected using aqueous ammonia solution at ambient temperature overnight, followed by purification using NAP-25 column, RP-HPLC and denaturing PAGE (see Figure S2). The porphyrin-DNA is isolated as free base porphyrin due to loss of zinc during acid detritylation in the SPS.

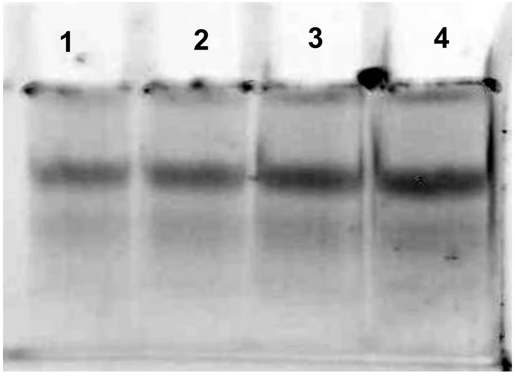

Figure S2. Preparative gel electrophoretic purification of tetra-phenyl porphyrin-modified DNA oligonucleotides generated by solid-phase oligonucleotide synthesis. 10 nmole each were loaded onto a 1 mm-thick 16% polyacrylamide gel with 200 μL loading slots. The gel was run at 180 V for 1 h. The bands were visualized by excitation at 424 nm. Lanes 1 and 2, porphyrin-modified DNA oligonucleotide A-TPP; lanes 3 and 4, B-TPP (for sequences of oligonucleotides see Table S1).

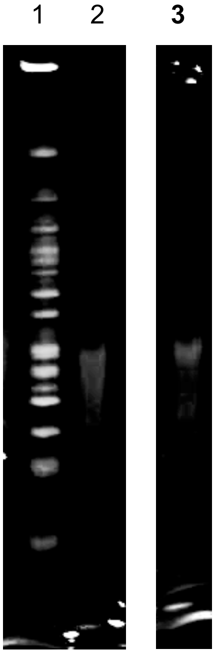

Figure S3. Analytical 16% PAGE of the gel-purified porphyrin-tagged oligonucleotide B-TPP. (A) The porphyrin-DNA migrates between the 300 and 350 marker bands. Lanes 1 & 2, ethidium bromide-stained gel. Lane 1, 50 bp marker. Lane 2, B-TPP. Lane 3, fluorescence-image of non-stained gel.

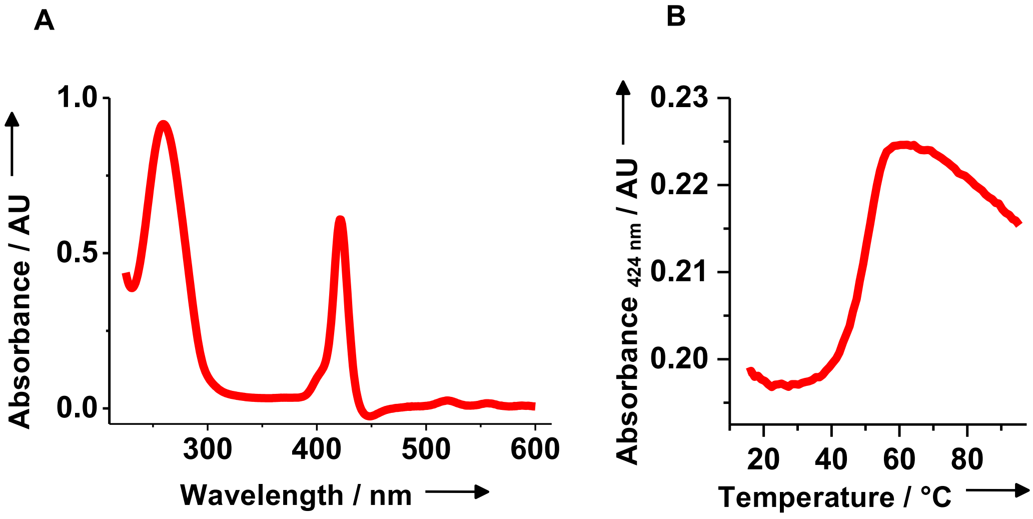

Figure S4. Spectroscopic analysis of porphyrin DNA oligonucleotide A-TPP using (A) UV-Vis absorption and (B) and porphyrin Soret band UV melting profile.

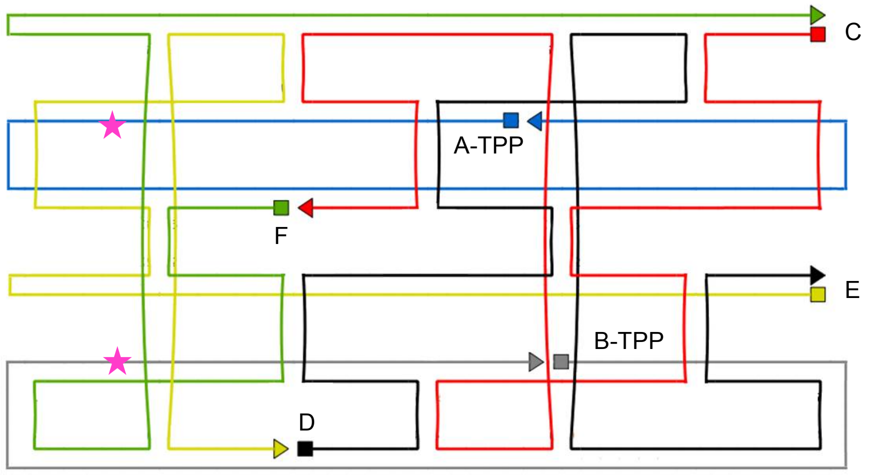

Figure S5. Map of the DNA nanobarrel composed of 6 DNA strands labeled by letters (Table S1). The pink stars indicate the base positions which carry a TPP porphyrin tag.

Table S1. Sequences of DNA oligonucleotides for assembling the porphyrin DNA-nanopore

ID	Sequence
A-TPP	TTATAAGGGATTTTGCCGATTT**P**GGAATTTTACAGGATTTTCGCCT GCTGGGGCAAACCAGCGTGGACCGCTTTTTTGGCTATTCTTTTGAT
B-TPP	GGCGCCCAATACGCTTTTTCCCCGCGCGTTGGCCGATTCATTAATGC AGCTGGCACGACATTTTTCTC**P**CTGGTGAAAAGAAAAACCACCCT
C	TGTTCCAAATAGCCAAGCGGTCCACGCTCCCTGAGGGGCGCC AGGGTGGGAATCGGACAAGAGTCCACTAAAATCCCCCCAGCA
D	CATTAATTTTTTCTCCTTCACCGCCTGGGGTTTGCTTATAAA TCAAAAGGTTTGGACCAACGCGCGGGGAGCGTATTAGAGTTG
E	CAACTCTCTCAGGGCCAGGCGGTGAAGGGCAATC*A*G*C*T*G*TTGTTTTCAA* C*A*G*C*A*T*C*C*TGTTTC*C*G*A*A*A*TCGGCATTAAAG*A*C*CAGCTG
F	GGCGAA*A*T*GATTGCTTTCAC*C*A*G*T*G*AGATGT*C*G*T*G*A*C*G*T *GGATTTTTCC*A*C*G*T*T*CTTTAATAGTGGACTCTTGTTCCAAACTGGAACA

The sequences start with the 5′ terminus. **P** indicates a deoxy-uridine nucleotide carrying a tetra-phenyl porphyrin modification. * signifies a phosphorothioate (PPT) modification. This modification was included in the original nanopore design to be comparable with our previous nanopore work [4] and to provide a back-up in case the porphyrin tags were not sufficient to insert the pore into the bilayer. In this eventuality, the PPT would have been chemically modified with an ethyl group to mask the negative charge.[4] The ethyl-groups would have formed a hydrophobic belt at the left end of the pore (Figure S5). However, this was not necessary as the native porphyrin-nanopore inserted at pH 8.0 where PPT is negatively charged and behaves as a regular phosphate group. Indeed, pores with non-alkylated PPT groups do not insert into lipid bilayers.[4] All experiments in this manuscript were carried out using a fully negatively charged porphyrin-nanopores in a buffer at pH 8.0.

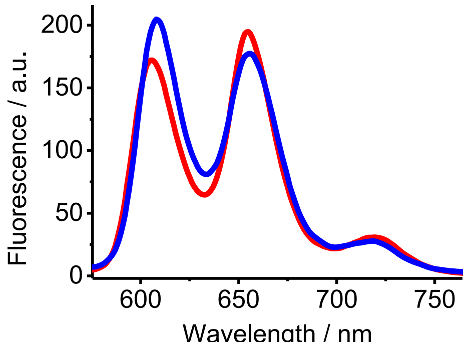

Figure S6. Fluorescence emission spectrum of zinc-porphyrin nanopores before (red) and after (blue) incubation with liposomes using an excitation wavelength of 426 nm. The concentration of porphyrin-nanopores was 0.5 µM and the original lipid concentration of DPhPC GUV liposomes was 500 µM. The porphyrins were metallated with zinc using a published protocol.[5]

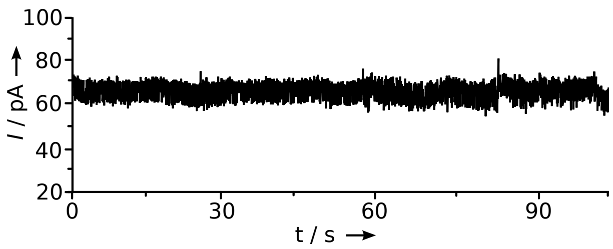

Figure S7. Ionic current trace of membrane-inserted porphyrin-tagged DNA nanopores at 100 mV in 1 M KCl, 50 mM Tris, pH 8.0 acquired at a filtering frequency of 2 kHz and a sampling rate of 10 kHz. The current level corresponds to three pores and remains stable for more than 90 s implying stable membrane insertion.

References:

[1] L.-A. Fendt, I. Bouamaied, S. Thoeni, N. Amiot, E. Stulz, *J. Am. Chem. Soc.* **2007**, *129*, 15319–15329.

[2] L. S. Bleicher, N. D. P. Cosford, A. Herbaut, J. S. McCallum, I. A. McDonald, *J. Org. Chem.* **1998**, *63*, 1109-1118.

[3] L. S. Bleicher, N. D. P. Cosford, A. Herbaut, J. S. McCallum, I. A. McDonald, *J Org Chem* **1998**, *63*, 1109-1118.

[4] J. Burns, E. Stulz, S. Howorka, *Nano Lett.* **2013**, *13*, 2351-2356.

[5] T. Nguyen, A. Brewer, E. Stulz, *Angew. Chem. Int. Ed.* **2009**, *48*, 1974-1977.
